# The Study of Exosomes-Encapsulated mPEG-PLGA Polymer Drug-Loaded Particles for Targeted Therapy of Liver Cancer

**DOI:** 10.1155/2022/4234116

**Published:** 2022-09-17

**Authors:** Jiantao Mo, Xuanbo Da, Qiaoxin Li, Jingjing Huang, Le Lu, Hongwei Lu

**Affiliations:** ^1^Department of General Surgery, Second Affiliated Hospital of Xi'an Jiaotong University, Xi'an 710004, Shaanxi, China; ^2^Department of Hepatobiliary Surgery, First Affiliated Hospital of Xi'an Jiaotong University, Xi'an 710061, Shaanxi, China; ^3^Center of Gallbladder Disease, Shanghai East Hospital, Institute of Gallstone Disease, School of Medicine, Tongji University, Shanghai 200092, China

## Abstract

The emergence of targeted drugs brings hope to patients with advanced liver cancer. However, due to the complex and diverse environment in the human body, the overall response rate of targeted drugs is not high. Therefore, how to efficiently deliver targeted drugs to tumor sites is a major challenge for current research. The project intends to construct mPEG-PLGA nanoparticles loaded with Sora and encapsulate them with exosomes for targeted therapy of hepatocellular carcinoma. mPEG-PLGA drug-loaded nanoparticles were prepared by the dialysis method and characterized by TEM and DLS. The obtained nanoparticles were incubated with the exosomes of liver cancer cells, and the exosomes-encapsulated drug-loaded nanoparticles (Exo-Sora-NPs) were obtained under pulsed ultrasound conditions, and they were characterized by Western blot, transmission electron microscopy (TEM), and dynamic light scattering (DLS). The toxic effect of Exo-Sora-NPs on liver cancer cells was detected by the CCK-8 experiment. The uptake efficiency of nanoparticles by liver cancer cells was detected by a confocal microscope. The accumulation and infiltration depth of nanomedicine in liver cancer tissues were observed by confocal microscope on frozen sections of liver cancer tissue after the H22 liver cancer subcutaneous tumor transplantation model was constructed. The tumor size, body weight, pathology, and serology analysis of mice were measured after administration. The mPEG-PLGA polymer drug-loaded particles encapsulated by exosomes have high targeting ability and biosafety. To a certain extent, they can target the drug to the tumor site with a smaller systemic response and have a highly effective killing effect on the tumor. Nanodrug-loaded particles encapsulated by exosomes have great potential as drug carriers.

## 1. Introduction

Hepatocellular carcinoma (HCC) has a high incidence and mortality rate and a short 5-year survival period, which has become a major hidden danger to human health and safety [1]. The emergence of molecular targeted drugs represented by Sorafenib (Sora) has brought new hope to liver cancer patients [2]. However, due to the complexity of the human body environment and immune microenvironment, a large part of the drug is consumed in the circulation after entering the human body, and the concentration of the drug reaching the tumor is very low [3, 4], resulting in a low overall response rate. Therefore, it shows how to effectively avoid surveillance by the immune system, escape the capture of the reticuloendothelial and mononuclear macrophages, and efficiently deliver targeted drugs to the tumor is a problem that needs to be solved urgently.

The nano drug delivery system which has unique advantages, such as enhanced retention effect, surface that can be coupled with targeted molecules, and drug codelivery, is very beneficial for targeted therapy of HCC [5]. However, due to its complex synthesis process to achieve coupling of targeting moieties, it has potential toxicity and side effects, and because of the existence of immune elimination, it is difficult to highly accumulate in tumor tissues, penetrate deep into tumor tissues, and be taken up by tumor cells in large amounts, thus limiting its role in nano-delivery [6–8]. At present, bioprocessed nanoparticles based on biofilms are widely used in tumor treatment [9, 10].

Exosomes originate from intracellular lysosomes, which contain a variety of proteins, polypeptides, and RNAs, and their morphology is a double-concavedisc-shaped vesicle structure with a diameter of 30–200 nm [11]. In recent years, exosomes have performed well as a nano-scale natural carrier to deliver specific therapeutic drugs (such as biomolecules or nanoparticles with thermotherapy capabilities) to target cells due to their wide sources, low immunogenicity, and high homology with parent cell membranes [12–14]. At present, exosomes have been used as endogenous drug carriers for the treatment of liver cancer and inflammatory diseases at home and abroad [15], but there are few studies on using exosomes to encapsulate nanodrug-carrying materials for drug delivery [16].

Biodegradable block copolymers occupy an important position in nanocarriers due to their efficient drug loading rate, good biocompatibility, and high bioavailability [17, 18]. Among them, mPEG-PLGA is currently widely used [19–21]. Based on this, we use exosomes as carriers, encapsulate sora-loaded polymer nanoparticles, and take advantage of the low immunogenicity, “homing” characteristics [22], and good biocompatibility of exosomes to target drugs to the inside of liver cancer cells and kill tumor cells more efficiently without causing systemic reactions.

## 2. Materials and Methods

### 2.1. Materials

mPEG_4K_-PLGA_24K_ (50 : 50) was purchased from Jinan Daigang Biotechnology Co., Ltd. (Jinan, China). Sora was obtained from MedChemExpress (NJ, USA). Coumarin6 (Standard, HPLC ≥98.0%) was obtained from Sigma-Aldrich (St. Louis, MO, USA). Dulbecco's Modified Eagle's Medium (DMEM), fetal bovine serum (FBS), penicillin, and streptomycin were provided by Gibco BRL/Life Technologies (Grand Island, NY, USA). The cell counting kit (CCK-8) assay was obtained from MedChemExpress (NJ, USA).The Anti-TSG101 antibody was purchased from Abcam plc (Cambridge, MA, USA). DIL, Hoechst 33258, and BCA protein quantification kit were purchased from Beyotime Biotechnology (Shanghai, China). DMF and DMSO were purchased from Sigma-Aldrich (St. Louis, MO, USA).

### 2.2. Animals and Cell Lines

Murine hepatocarcinoma cell line H22, and human hepatocarcinoma cell line Huh-7, MHCC97H were obtained from the Type Culture Collection of the Chinese Academy of Sciences (Shanghai, China). H22 cells were cultured in RPMI 1640 medium, and Huh-7, and MHCC97H were cultured in DMEM medium at 37°C in a cell incubator with 5% CO_2_. Six- to eight-week-old BALB/*c* mice (female) were purchased from the Experimental Animal Center of Xi'an Jiaotong University. A H22 mouse liver cancer subcutaneous tumor transplantation model was constructed by subcutaneously injecting 10^7^ H22 cells per mouse into the right shoulder of female BALB/*c* mice. All animal procedures were performed in accordance with the animal protocols approved by the Ethical Committee of the Second Affiliated Hospital, Xi'an Jiaotong University, Xi'an, China.

### 2.3. Preparation and Characterization of Sora-NPs and Exo-Sora-NPs

Nanoparticles loaded with sorafenib (Sora-NPs) are prepared by a dialysis method. Firstly, mPEG_4K_-PLGA_24K_ copolymer (50 : 50) was dispersed in dimethylformamide, and Sora was dispersed in dimethyl sulfoxide with a concentration of 10 mg/ml. Second, mPEG-PLGA and Sora solutions were added to a 50 ml centrifuge tube at a volume ratio of 5 : 1, and then five times the volume of Milli-Q ultrapure water (Millipore, 18.2 MU, Bedford, MA) was added under vigorous stirring. After stirring for about 10 min, the mixture was dialyzed against ultrapure water in a dialysis bag (Spectra/Por®, Float-A-Lyzer, molecular weight cut-off (MWCO) = 1.5 kDa) to remove the organic solution. The unencapsulated sorafenib was removed by centrifugation at 5000*g* for 10 min, and the obtained nanoparticles were concentrated with an Amico filter device (Millipore) with a MWCO of 100 kDa for further use. The same procedure preparing Sora-NPs was used to prepare unloaded NP except that Sora was not added.

The preparation of exosomes is mainly accomplished by exosome purification kits (Umibio, Shanghai, China). In brief, huh-7 cells were grown in DMEM supplemented with 10% FBS and 1% penicillin-streptomycin mixture. When the density of cells came to 60%, the old medium was discarded. The cells were washed three times with sterile PBS, replaced with serum-free high-glucose DMEM medium and cultured for 48 h. Then the cell supernatant was collected and centrifuged to remove dead cells and cell debris. The ECS solution was added to the cell supernatant and mixed by inversion overnight at 4°C. The pellet was obtained by centrifugation at 10000*g* for 60 min. The pellet was then resuspended in PBS and transferred to an EPF column for purification to obtain exosomes.

Exo-Sora-NPs were prepared by pulsed ultrasound. The Eppendorf tube containing a mixed solution of Sora-NPs and exosomes (100 *μ*g) was inserted into the float, suspended in the ultrasonic cleaner. Then, the ultrasonic power was adjusted to 40% (200 W), and turned on and off for 15 s, respectively. After three repetitions, the Eppendorf tube was taken out and placed on ice in an ice bath for 2 min. The abovementioned operation was repeated three times. When the process ended, the Eppendorf tube was placed in a constant temperature water bath. The temperature was adjusted to 37°C, and incubated for 1 h to restore the stability of the exosomal membrane. After the constant temperature incubation in the water bath, the mixed solution of the drug and exosomes was transferred to a 100 kDa ultrafiltration tube, and the excess drug molecules were centrifuged at 4500*g* for 15 min.

DLS and TEM were used to detect the size, potential, and morphology of the obtained nanoparticles. Malvern Dispersion Technology Software 7.0.2 was used to analyze the data.

### 2.4. Determination of the Drug Loading (DL)

1 mg of lyophilized Sora-NPs was dispersed in 1 ml of ultrapure water. Its absorbance was calculated by a UV spectrophotometer. Subsequently, the encapsulation efficiency was calculated according to Sora's standard curve and the total mass of Sora-NPs.

(1) $$\begin{array}{c} DL\left(\%\right)=\frac{\text{Amount of Sora in solution}}{\text{Weight of nanoparticles}}*100\%. \end{array}$$

### 2.5. Identification of Surface Markers before and after Drug Loading of Exosomes

The protein content of purified exosomes and Exo-Sora-NPs were determined by BCA protein concentration determination kit and then subjected to western blot analysis. Anti-TSG101 was used as primary antibodies.

### 2.6. Confocal Microscopy to Investigate the Endocytosis of Exo-Sora-NPs by Liver Cancer Cells

Huh-7 cells were seeded onto twenty-four well plates overnight at a density of 2 × 10^5^ cells per well. Afterward, cells were incubated with C6-NPs, and Exo-C6-NPs at different C6 concentrations for 4 h. A 4% paraformaldehyde solution was added to each well to fix the liver cancer cells. After washing with PBS, the nucleus and cell membrane were labeled with Hoechst 33258 solution and DIL, respectively. The slide was removed and observed by a confocal laser.

### 2.7. Cytotoxicity Investigation In Vitro Using CCK-8 Assay

To investigate the toxicity of Exo-Sora-NPs on liver cancer cells, Huh-7 and MHCC97H cells were seeded in a 96-well plate at a density of 1 × 10^4^ cells per well overnight and then treated with free Sora, blank NPs, Sora-NPs, or Exo-Sora-NPs at different Sora concentrations for 24 h. Subsequently, the cells were treated with 10 *μ*L CCK-8 reagent for 2 h and the absorbance at 450 nm was measured using a microplate reader.

### 2.8. Study on Deep Tumor Penetration Behavior in H22 Subcutaneous Tumor Mice

When the tumor volume grew to about 250 mm^3^, free C6, C6-NPs and Exo-C6-NPs with a C6 concentration of 0.5 mg/kg were injected into the tail vein. Twenty-four hours later, the mice were sacrificed by cervical dislocation, and the tumor tissues were taken out for frozen section processing. The CD34 antibody (Abcam, 81289, diluted to 1 : 600) was incubated at 37°C for 30 min to label the blood vessels of tumor sections. A confocal microscope was used to detect C6 green fluorescence and CD34 red fluorescence on the slices at 360/477 nm and 590/617 nm, respectively.

### 2.9. The Inhibitory Effect and Biosafety of Exosomes-Encapsulated Nano Drug Delivery System on H22 Tumor-Bearing Mice

10^7^ H22 liver cancer cells were injected subcutaneously into the right shoulder of BALB/*c* mice (14–16 g) to construct a mouse subcutaneous tumor transplantation model. When the tumor volume grew to about 250 mm^3^, the mice were divided into 4 groups, with 5 mice in each group. PBS, Sora, Sora-NPs, or Exo-Sora-NPs were injected into the tail vein (final concentration of Sora is 10 mg/kg). The tumor size and the mouse body weight were measured at a fixed time every two days. On the 14th day after administration, the mice were sacrificed by the cervical dislocation method, the tumor tissue was taken out, washed, dried, weighed, and photographed for subsequent H&E staining. At the same time, blood was collected from the mice after 14 days of action on the drug-loaded nanoparticles, and the serum was collected by centrifugation for liver and kidney function tests. The heart, liver, spleen, lung, kidney, and other major organs were collected, cleaned, and photographed, and fixed with 4% paraformaldehyde for subsequent H&E staining.

### 2.10. Statistical Analysis

The data in the experiment were all analyzed by Prism software (version 9.0). The data were repeated 3 times and the average value was calculated. One-way analysis of variance was used to analyze the data between different groups. *P* < 0.05 was regarded as statistically different. In the figure, “^*∗*^” means *P* < 0.05, and “^*∗∗*^” means *P* < 0.01.

## 3. Results

### 3.1. Synthesis and Characterization of Membrane-Coated Sora-NPs

**Figure 1:**
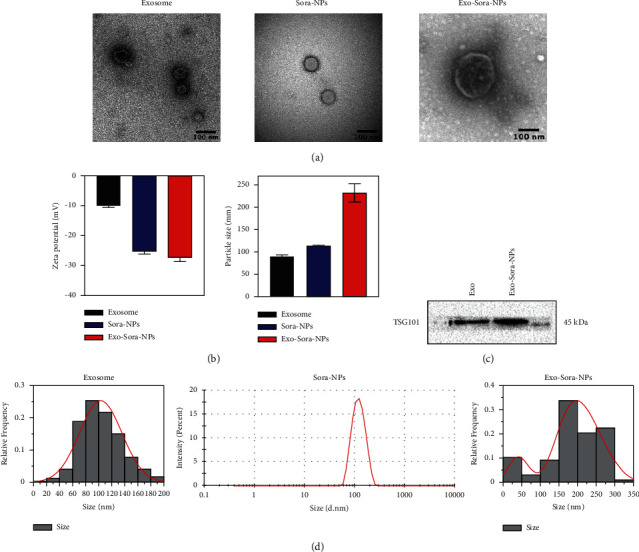
The physicochemical characteristics of Exo-Sora-NPs. (a) Sora-NPs and the morphology of exosomes before and after loading under TEM. (b) Exosome, Sora-NPs, Exo-Sora-NPs particle size and potential change graph (*n* = 3). (c) Western blot detection of exosomal-specific protein expression. (d) DLS measurement of the particle size of nanoparticles and exosomes before and after drug loading.

After exosomes of Huh-7 cells were incubated with Sora-NPs under the condition of pulsed ultrasound, we collected the internalized Sora-NPs (Exo-Sora-NPs) by centrifugation. Huh-7 exosomes present a disc-shaped vesicle structure, and the morphology of the exosomes loaded with Sora-NPs has not changed from the TEM (Figure 1(a)). The zeta-potential of Exo-Sora-NPs and Sora-NPs were −27.29 ± 1.46 mV and −25.21 ± 0.99 mV, respectively (Figure 1(b)). The drug loading of Sora in Sora-NPs and Exo-Sora-NPs were 2.4% and 1.9%. Furthermore, Western blot experiments further showed that the exosome obtained by the kit method, exosome biomarker TSG101 was also detected in Exo-Sora-NPs, confirming the presence of exosomes in Exo-Sora-NPs (Figure 1(c)). DLS analysis showed that the size of Exo-Sora-NPs and Sora-NPs was 231.79 ± 20.09 nm and 114.67 ± 0.55 nm, and the corresponding PDI was 0.145 ± 0.032 and 0.06 ± 0.01, respectively (Figure 1(d)).

### 3.2. Cellular Uptake and Cytotoxicity of Biomimetic NPs

**Figure 2:**
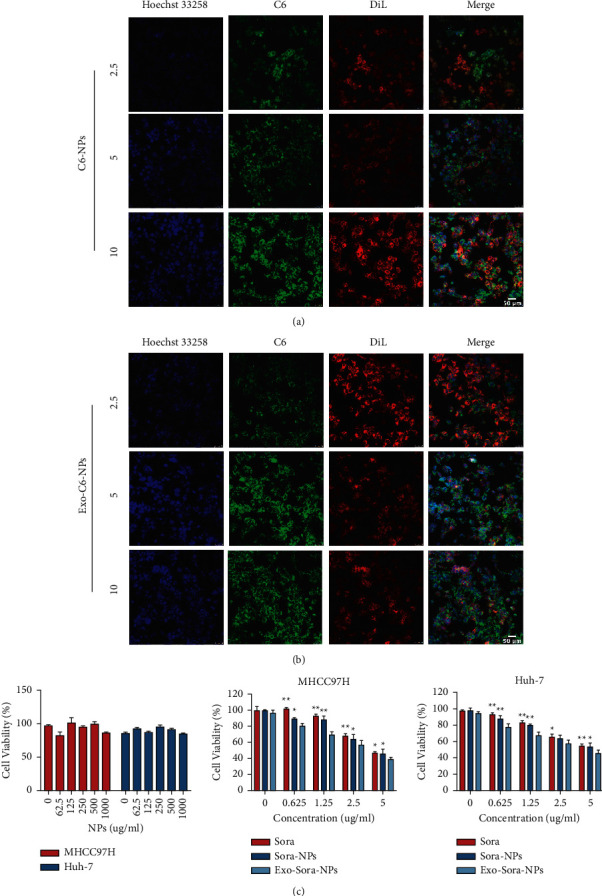
Enhanced cellular uptake and anti-tumor properties of Exo-Sora-NPs. (a), (b) The uptake of Exo-C6-NPs by Huh-7 cells under a concentration gradient of a confocal microscope. (c) Toxic effects of different concentrations of blank NPs, Sora, Sora-NPs, Exo-Sora-NPs on Huh-7 cells and MHCC97H cells (*n* = 3). Compared with the Sora and Sora-NPs groups, Exo-Sora-NPs significantly inhibited tumor growth. ^*∗*^indicates *P* < 0.05, ^*∗∗*^indicates *P* < 0.01.

To evaluate whether Exo-Sora-NPs possess cross-reactive cellular uptake and cytotoxicity, human hepatocarcinoma Huh-7 cells were treated with C6-NPs or Exo-C6-NPs. DIL and C6 were used to label exosome membranes and Sora-NPs respectively to judge that NPs were located in exosomes by observing the fluorescence colocalization of the two (Supplementary Figure 1). Consistently, Exo-C6-NPs showed higher internalization into Huh-7 cells compared with C6-NPs (Figures 2(a) and 2(b)). Sora, Sora-NPs, and Exo-Sora-NPs inhibited the proliferation of Huh-7 cells and MHCC97H cells in a concentration-dependent manner. When the effective concentration of Sora came to 5 ug/ml, the inhibition rate of the Exo-Sora-NPs group on Huh-7 cells reached 54.42%, which was 1.19 and 1.17 times that of the free Sora and Sora-NPs groups, respectively. The inhibition rate of the Exo-Sora-NPs group on MHCC97H cells reached 61.09%, which was 1.15 and 1.13 times that of the free Sora and Sora-NPs groups, respectively, with significantly statistical differences (Figure 2(c)). These results suggest that Exo-Sora-NPs have strong cellular uptake and cytotoxicity against Huh-7 and MHCC97H cells.

### 3.3. Enhanced Tumor Accumulation and Penetration

**Figure 3:**
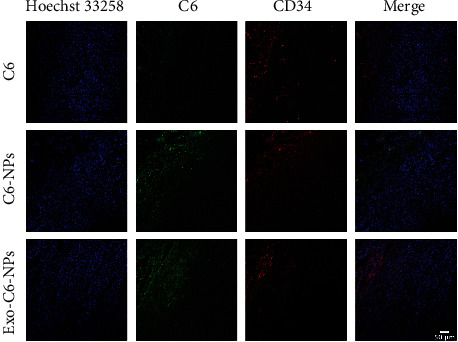
Enhanced tumor accumulation and penetration of Exo-C6-NPs. Immunofluorescence staining of frozen sections of mouse tumor tissues treated with C6, C6-NPs, or Exo-C6-NPs. Bar = 50 *μ*m.

Free C6, C6-NPs, and Exo-C6-NPs were injected into tumor-bearing mice through the tail vein, and the tumor tissues were taken out and frozen sectioned after 24 h. Hoechst 33258 and CD34 marked the nucleus and the tumor blood vessels, respectively. The results of confocal microscopy showed that the red tumor blood vessels were mainly concentrated on the surface of the tumor tissue and the nanoparticles infiltrated the tumor tissue layer by layer. The green fluorescence of the free C6 group was very weak and localized on the surface of the tumor. The fluorescence of the C6-NPs group increased slightly, but it was still confined to the surface. The fluorescence of the Exo-C6-NPs group was significantly enhanced, and the depth of infiltration was significantly stronger than that of the C6-NPs group and the free drug group. The abovementioned results verify that Exo-C6-NPs have an obvious deep penetration ability of tumors (Figure 3).

### 3.4. Excellent Anticancer Ability

**Figure 4:**
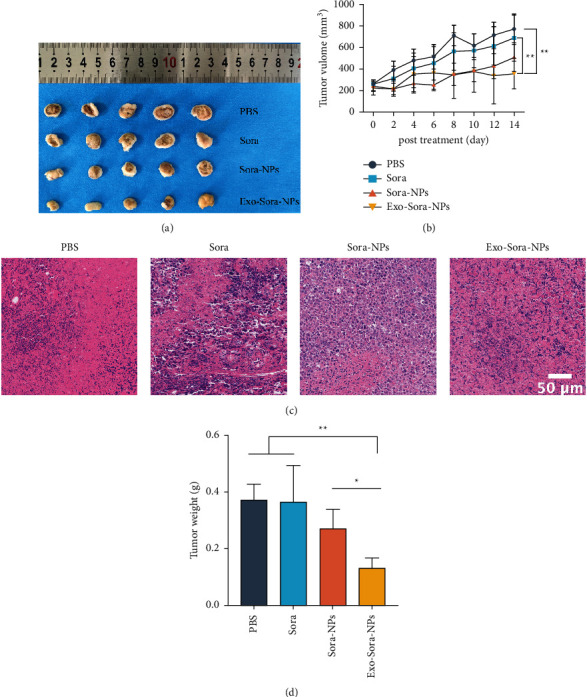
Enhanced antitumor properties of Exo-Sora-NPs. (a) Optical image of tumor tissue in H22 tumor-bearing mice. (b) Changes in tumor volume, (c) tumor pathology and (d) tumor weight. The data are shown as mean ± SD (*n* = 5). “^*∗*^” indicates *P* < 0.05, “^*∗∗*^” indicates *P* < 0.01.

As shown in Figure 4(a), the tumor tissue of the Exo-Sora-NPs group was significantly reduced compared with the PBS group, the free Sora group, and the Sora-NPs group under the light microscope, showing a significant inhibitory effect on the tumor tissue. Tumor growth curve results showed that Exo-Sora-NPs significantly inhibited tumor growth and its tumor suppression effect was significantly better than the same concentration of the free Sora group and Sora-NPs group in Figure 4(b). H&E staining results showed that the number of cells in the free Sora group decreased (Figure 4(c)). The nuclei of tumor cells treated with Sora-NPs shrank, divided, had irregular shapes, and showed signs of necrosis. In the Exo-Sora-NPs group, no obvious cell structure was found in many tumor tissues, and the degree of necrosis was more obvious, proving that it has a stronger inhibitory effect on tumors.  Figure 4(d) shows that the weight of the tumor in the Exo-Sora-NPs group was significantly reduced, and it had stronger antitumor effects compared with the PBS group, free Sora group, and Sora-NPs group. After the administration was completed, the weighing results of the removed tumor tissue also showed that Exo-Sora-NPs significantly inhibited tumor growth. The tumor volume in the PBS group increased significantly, reaching 3 times the initial tumor size at the end of 14 days. Compared with the PBS group, the free Sora group inhibited the growth of the tumor, but the tumor volume increased more obviously, and the tumor grew to 2.6 times the initial tumor size. In the Sora-NPs group, a higher drug concentration was formed in the tumor at the initial stage, which had a significant inhibitory effect on the tumor. However, due to the metabolism of drugs in the body, the tumor volume continued to increase after 2 days and grew to 2.1 times the initial tumor size. The mice treated with the Exo-Sora-NPs group showed stronger antitumor effects, and the volume was only 1.6 times the size of the initial tumor. The above data indicate that Exo-Sora-NPs drug-loaded nanoparticles have a strong inhibitory effect on tumor growth.

### 3.5. Biosafety Investigation of Exo-Sora-NPs

**Figure 5:**
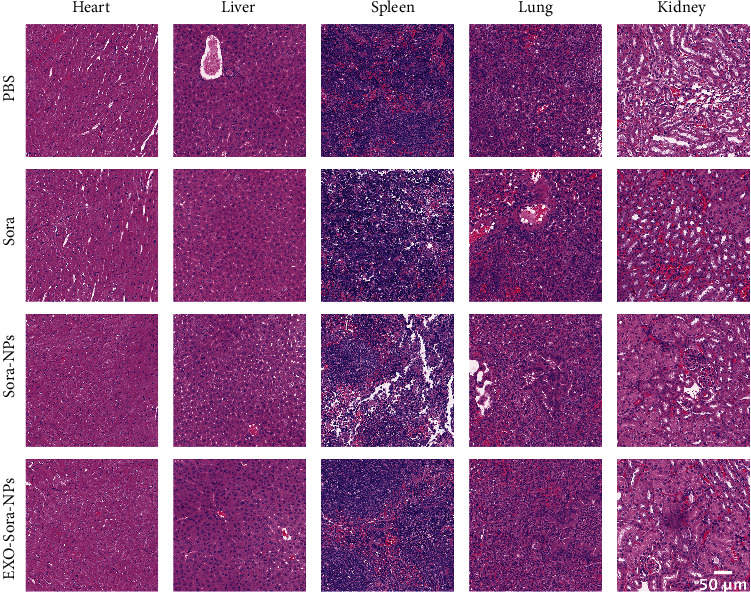
Excellent biosafety of Exo-Sora-NPs. H&E-stained sections of mouse heart, liver, spleen, lung, and kidney treated with PBS, Sora, Sora-NPs, or Exo-Sora-NPs. Bar = 50 *μ*m.

**Figure 6:**
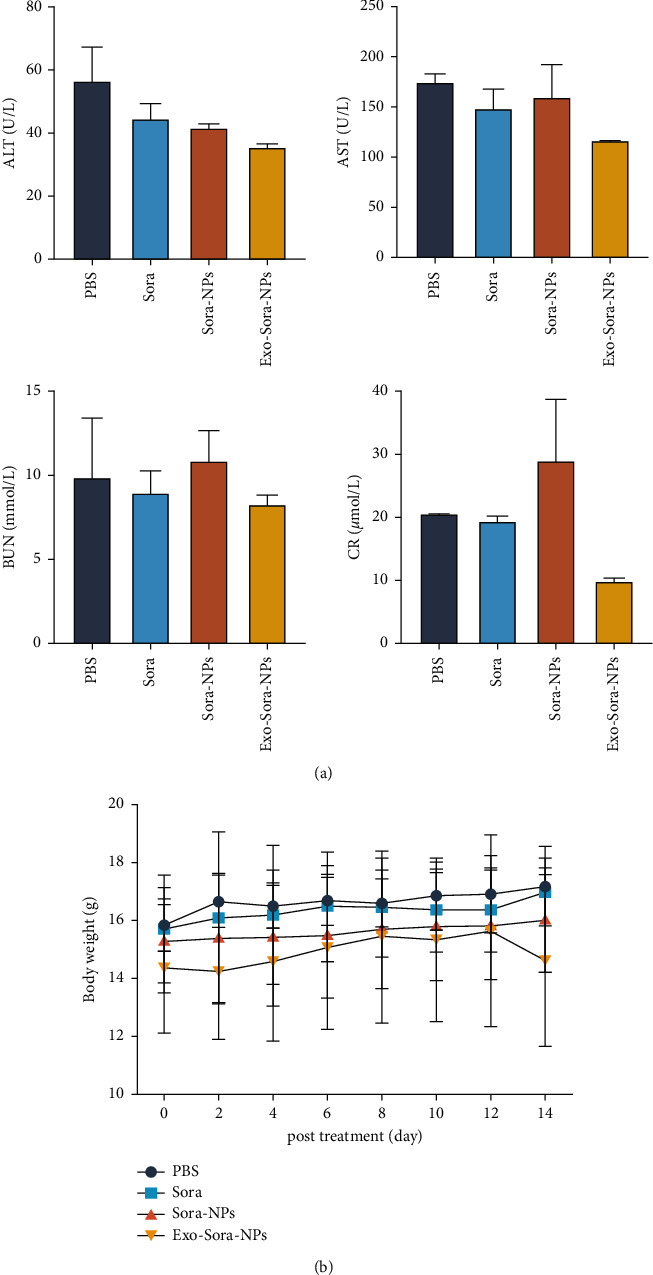
Excellent biosafety of Exo-Sora-NPs. (a) Serological analysis of H22 tumor-bearing mouse model. (b) Body weight in H22 tumor-bearing mice.

The H&E slices of the PBS group, Sora group, Sora-NPs group, and Exo-Sora-NPs group showed that the main organs were in the complete structure, the cells were arranged regularly, and no significant cell necrosis is found in Figure 5.  Figure 6(a) examines the safety from the perspective of blood biochemistry, and the liver and kidney functions of each group were within the normal range.  Figure 6(b) shows that all body weights of the control group and the experimental group maintained small fluctuations within 14 days. The slight difference indicates that the exosomal drug-loaded particles have not significantly affected the functions of important organs such as the liver and kidney. Therefore, Exo-Sora-NPs have good biological safety.

## 4. Discussion

With the continuous development of science and technology, the treatment of HCC is constantly updated but there is still a lack of effective methods for the treatment of advanced HCC [23]. The emergence of targeted therapy has brought new hope to patients with advanced HCC, in which Sora has been approved as first-line targeted therapy [24, 25]. Since the liver is an immune preferential organ with special immunosuppressive cells, the therapeutic effects of Sora and chemotherapy in the past were unsatisfactory [26]. Furthermore, due to the pharmacokinetic characteristics of the body and the complexity of the immune microenvironment, it is difficult for targeted drugs to form a sufficient drug concentration in the tumor, so the overall response rate is not high, and even accompanied by immune side effects [27].

Simple drug-loaded nanoparticles activate a stronger immune elimination effect due to the modification of functional ligands to enhance their targeting ability [28]. In addition, due to the genetic and phenotypic heterogeneity of tumors, their effects on drug delivery are not ideal [29]. The rise of biofilm-based drug-loaded nanoparticles has solved these problems, and exosomes play an important role as endogenous carriers [30–32]. At present, exosomes have successfully delivered small-molecular chemotherapeutic drugs, genes, and anti-inflammatory drugs to target tissues, and achieved good results [33].

In the present study, we developed an exosome-sheathed mPEG-PLGA to load Sora for efficient HCC targeting and killing. Exo-Sora-NPs not only exhibited enhanced tumor accumulation and penetration but also had strong cross-reactive cellular uptake against HCC, as evidenced by the fact that Exo-Sora-NPs are efficiently internalized into Huh-7 cells. Sora-NPs and Exo-Sora-NPs showed obvious cytotoxicity to Huh-7 and MHCC97H cells. The strong cross-reactive cellular uptake of Exo-Sora-NPs can overcome the obstacles of requiring specific markers for targeting HCC. Therefore, Exo-Sora-NPs efficiently integrated all features to eradicate HCC, generating remarkable anticancer activity in H22 tumor-bearing BALB/*c* mice. No significant toxicity of Exo-Sora-NPs was observed in tumor-bearing mice by serological and histopathological analysis.

All in all, we had successfully developed biocompatible exosome-sheathed NPs for targeted HCC therapy. Exo-Sora-NPs are produced from exosomes incorporated with Sora-NPs by pulsed ultrasound. Following intravenous injection, Exo-Sora-NPs exhibit enhanced tumor accumulation, tumor penetration, and cross-reactive cellular uptake by bulk cancer cells, resulting in augmented in vivo Sora enrichment in total tumor cells. Exo-Sora-NPs further demonstrate significant cross-reactive anticancer activity in subcutaneous transplantation tumor models. Our study clearly demonstrates that exosome-biomimetic nanoparticles have potential as drug carriers to improve the anticancer efficacy.

## Data Availability

The datasets generated during and/or analyzed during the current study are not publicly available but are available from the corresponding author on reasonable request.

## Supplementary Materials

Supplementary Materials Supplementary Figure 1: The colocalization of C6-NPs and exosomes.
