# Supplementary figures and images for: The Study of Exosomes-Encapsulated mPEG-PLGA Polymer Drug-Loaded Particles for Targeted Therapy of Liver Cancer
Source: J Oncol. 2022 Sep 17;2022:4234116. doi: 10.1155/2022/4234116 (PMC9509232; doi:10.1155/2022/4234116)

C6-NPs

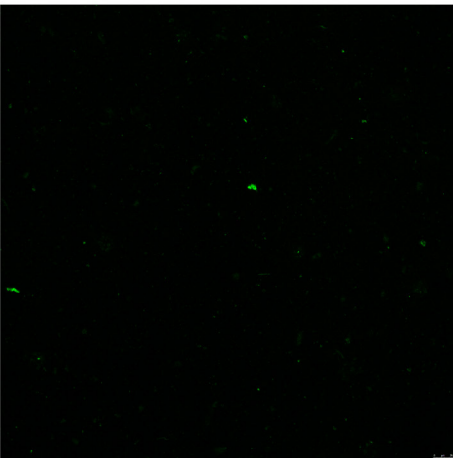

DiL

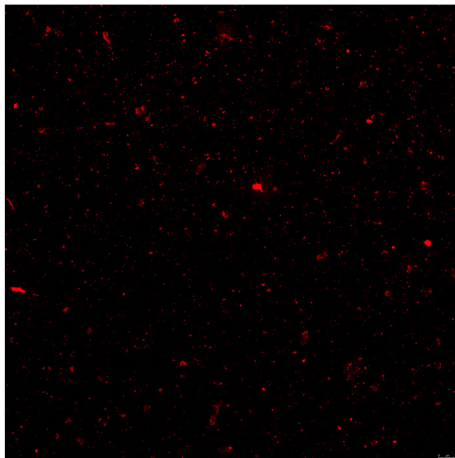

Exo-C6-NPs

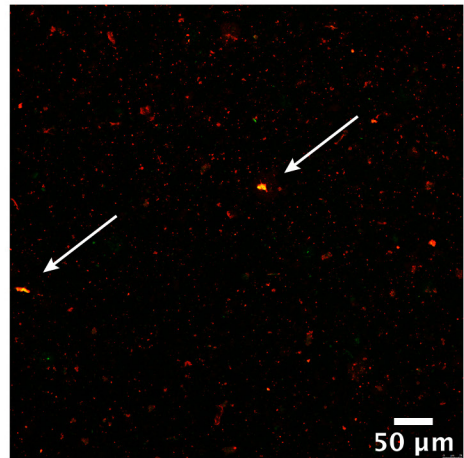

Supplementary Figure 1 The colocalization of C6-NPs and exosomes.

Supplement: Supplementary Materials — Supplementary Figure 1: The colocalization of C6-NPs and exosomes. [file 4234116.f1.pdf]
